# Essential Oil Composition of Stems and Fruits of *Caralluma europaea* N.E.Br. (Apocynaceae)

**DOI:** 10.3390/molecules15020627

**Published:** 2010-01-27

**Authors:** Pietro Zito, Maurizio Sajeva, Maurizio Bruno, Antonella Maggio, Sergio Rosselli, Carmen Formisano, Felice Senatore

**Affiliations:** 1 Dipartimento di Scienze Botaniche, Università degli Studi di Palermo, Via Archirafi 38, I-90123 Palermo, Italy; 2 Dipartimento di Chimica Organica, Università degli Studi di Palermo, Viale delle Scienze, Parco d’Orleans II, I-90128 Palermo, Italy; 3 Dipartimento di Chimica delle Sostanze Naturali, Università degli Studi di Napoli “Federico II”, Via D. Montesano, 49, I-80131 Napoli, Italy

**Keywords:** antimicrobial, Apocynaceae, *Caralluma europaea*, essential oils, semiochemicals

## Abstract

The essential oil of the stems and fruits of *Caralluma europaea* (Guss.) N.E.Br. (Apocynaceae) from Lampedusa Island has been obtained by hydrodistillation and its composition analyzed. The analyses allowed the identification and quantification of 74 volatile compounds, of which 16 were aromatic and 58 non-aromatic. Stems and fruits contained 1.4% and 2.7% of aromatic compounds respectively, while non-aromatic were 88.3% and 88.8%. Non-aromatic hydrocarbons were the most abundant compounds in both organs, followed by fatty acids. Data showed differences in the profiles between stems and fruits which shared only eighteen compounds; stems accounted for 38 compounds while fruits for 53. Fruits showed a higher diversity especially in aromatic compounds with twelve versus four in stems. Among the volatiles identified in stems and fruits of *C. europaea* 26 are present in other taxa of Apocynaceae, 52 are semiochemicals for many insects, and 21 have antimicrobial activity. The possible ecological role of the volatiles found is briefly discussed.

## 1. Introduction 

Volatile compounds with different ecological roles are widely produced by plants. The review by Dudereva *et al.* [1] indicates the different roles, such as pollinator attraction, defence from phytophagous and pathogenic microbes, allelopathy, seed dispersal, and thermotolerance, among others. There are few studies on the chemical composition of the genus *Caralluma*. The presence of pregnane steroids in several species of *Caralluma* has been reported in previous chemical studies [2,3,4,5] and it could indicate a systematical importance within the genus. On the other hand only a few species have shown the occurrence of flavonoids [6,7,8] and the only paper published on the chemical constituents of *Caralluma europaea* (Guss.) N.E.Br. is that of Meve and Heneidak [9]. Recently Formisano *et al.* [10] analysed the chemical volatiles composition by headspace of *C. europaea* and discussed their possible role in the biology of pollination linked to the sapromyiophilous syndrome typical of the Stapeliads. *Caralluma europaea* [= *Apteranthes europaea* (Guss.) Plowes] is a stem-succulent member of Apocynaceae – Asclepiadoideae, distributed in Egypt, S. Spain, Italy (Lampedusa Island), Libya, Tunisia, Algeria and Morocco [9]. It has quadrangular stems and forms large clumps up to 15–20 cm in diameter, flowers are red-brown with yellow stripes or strikes, 10–15 mm in diameter, and the corona is normally purplish [11]. Fruits are dehiscent follicles up to 20 cm long which at maturity release wind-dispersed seeds. To the best of our knowledge, no phytochemical studies on the essential oil of any species of *Caralluma* have been reported so far. In the present paper we present data on the essential oil composition of stems and fruits of *C. europaea* by hydrodistillation and compare the results with data available in scientific literature.

## 2. Results and Discussion

The analysis by GM/MS allowed the identification of 74 volatile compounds of which 16 aromatic and 58 non-aromatic (Table 1). Stems (S) and fruits (F) contained 1.4% and 2.7% of aromatic compounds respectively, while non-aromatic were 88.3% and 88.8%.

Non-aromatic hydrocarbons were the most abundant compounds in both organs, followed by fatty acids. The most abundant compounds were hentriacontane (S 9.5%; F 7.7%) nonacosane (S 8.3%; F 6.5%), heptacosane (6.1%; 9.9% F), tricosane (S 4.4%; F 7.3%), pentacosane (S 5.4%; F 6.5%) hexadecanoic acid (S 7.8%; F 9.6%), β-eudesmol (S 1.9%; F 5.4%), tetradecanoic acid (S 5.6%; F 0%) and (*Z,Z*)-9,12-octadecadienoic acid (S 5.2%; F 2.4%). Data showed differences in the profiles between stems and fruits which shared only eighteen compounds; stems accounted for 38 compounds while fruits for 53. Fruits showed a higher diversity especially in aromatic compounds with 12 compounds versus four in stems. Among the scarce literature available on volatiles in aerial parts of Apocynaceae, excluding flowers, some compounds have been found in leaves and stems of *Catharanthus roseus* [12,13] and in fruits of *Hancornia speciosa* [14] (Table 2).

Fifty-five volatiles found in stems and fruits of *C. europaea* are semiochemicals for many insects (Table 2). There are no synomones, which are usually related to pollinators and common in the flowers, while there are 28 attractants, 32 allomones, 21 pheromones and 49 kairomones. In literature there are several papers devoted to the highly specialized role of plant-animal interactions [1] and the semiochemicals present in *C. europaea* may play key roles in the adaptation of the species to its environment. 

**Figure 1:**
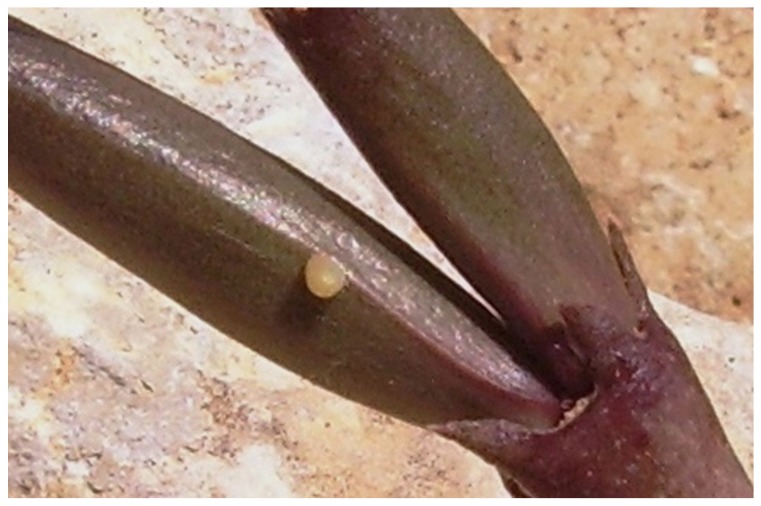
Egg of *Danaus chrysippus* on a fruit of *C. europaea* in Lampedusa Island (Photo by P. Zito).

**Figure 2:**
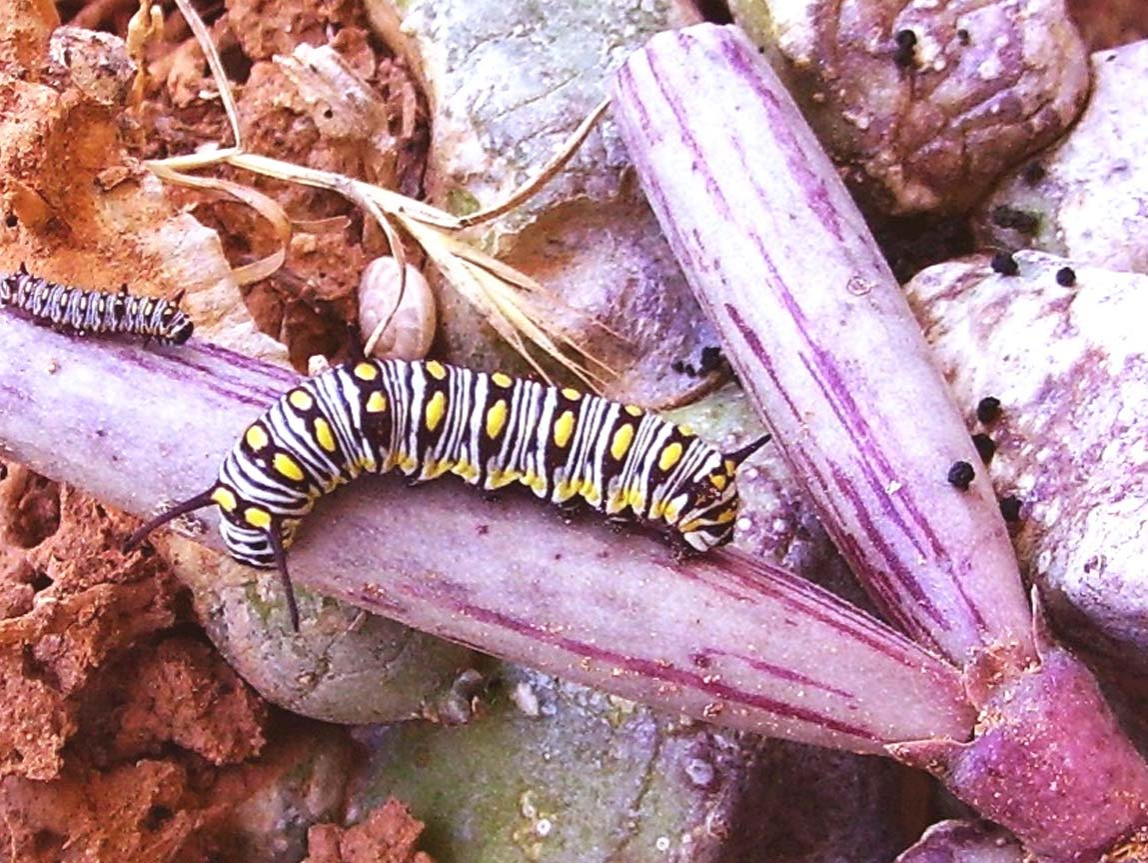
Larvae of *D. chrysippus* feeding on fruits of *C. europaea* in Lampedusa Island (Photo by P. Zito).

Plant semiochemicals are known to produce a wide range of behavioral responses in insects. Some insects sequester or acquire host plant compounds and use them as sex pheromones or sex pheromones precursors. Other insects produce or release sex pheromones in response to specific host plant cues, and chemicals from host plants often synergistically enhance the response of an insect to sex pheromones [17]. It is interesting to note that at least four of the most abundant volatiles found in *C. europaea* (pentacosane, hexacosane, heptacosane and hexadecanoic acid) are male pheromones for *Danaus chrysippus* [18], a butterfly whose larvae feed on plants rich in cardenolides like Apocynaceae and Moraceae [19]. In a recent paper Pisciotta *et al*. [20] observed that *D. chrysippus* in Lampedusa island oviposited only on the fruits of *C. europaea* (Figure 1) and that larvae fed on its fruits and stems (Figure 2). 

According to Reddy and Guerrero [17] the effects of host plants on pheromone behavior appear to be part of male strategies to maximize encounters with females as well as of female strategies to gain access to new feeding and oviposition sites. It is possible that the male pheromones guide the female of *D. chrysippus* to the ovipostion site and influences the feeding behavior of the larvae, thus having a negative effect on the fitness of the plant. Twenty one volatiles found in *C. europaea* are antimicrobial agents (Table 2) and fatty acids are among the main constituents. (*Z,Z*)-9,12-Octadecadienoic acid, tetradecanoic acid and hexadecanoic acid show antimicrobial activity against *Candida albicans, Clostridium welchii* and *Staphylococcus aureus* [21,22]. Walters *at al.* [23] also indicated that (*Z,Z*)-9,12-octadecadienoic acid has antifungal activities on the plant pathogenic fungi: *Rhizoctonia solani, Pythium ultimum, Pyrenophora avenae* and *Crinipellis perniciosa*. According to González-Lamothe *et al.* [24] plants are continuously in contact with different microorganisms, including viruses, bacteria and fungi many of which are pathogens that affect plant development, reproduction and ultimately yield production. 

## 3. Experimental

Plant material was collected in Lampedusa Island (Italy, 35°29’28” and 35°21’39” N – 12°30’54” and 12°37’55” E) from plants growing in the “Isola dei Conigli” area at an altitude of 100 m a.s.l. Stems and fruits were collected in April 2008, placed in paper bags and kept at 4 ± 1 °C for three hours before the hydrodistillation. Clones of the plants used are cultivated at the Botanical Garden of Palermo and a voucher specimen (N° PAL/MS/1112) was deposited in the Herbarium, Orto Botanico, Palermo, Italy. 

Air dried stems (23.64 g) and fruits (41.67 g) were hand-cut into small fragments and hydrodistilled in a Clevenger-type apparatus for three hours as previously described by Riela *et al.* [25]. The waxy oils were collected by *n*-pentane extraction, dried over anhydrous sodium sulphate and removal of the solvent. The essential oil yields was 0.38 mg (1.60%) and 4.90 mg (11.75%) for stems and fruits, respectively. The oil samples, characterized by a typical malodorous odour, were stored in a refrigerator at –10 °C until analysed. Analyses of essential oils were performed on a Perkin Elmer Sigma 115 gas chromatograph (GC) equipped with two different polarity-fused silica capillary columns: HP 5MS and HP Innowax, both 30 m long x 0.25 mm ID; 0.25 μm film thickness. Temperature program: initial temperature, 40 °C; hold 5 min; temperature rate 4 °C min^-1^, final temperature 250 °C; hold 30 min; column flow rate 1.0 mL He min^-1^; injector and detector temperatures 260 °C and 280 °C, respectively. Injection volume: 1.0 μL of diluted samples (1/100 v/v, in *n*-pentane) in the splitless mode. GC/MS analyses were performed on a Agilent 6850 Series II apparatus using an HP-5 fused silica capillary column (30 m long × 0.25 mm ID; 0.33 μm film thickness), connected to a quadrupole detector operating in electron impact (EI) mode at 70 eV; electron multiplier energy 2,000 V. Most constituents were identified by gas chromatography by comparison of their retention indices (R_i_) with either those of the literature [26,27] or with those of authentic compounds available in our laboratories. The retention indices were determined in relation to a homologous series of *n*-alkanes (C_8_–C_28_) under the same operating conditions. All the compounds were identified by comparison of their mass spectra on both columns with either those stored in NIST 02 and Wiley 275 libraries or with mass spectra from the literature [26,28] and a home made library. Component relative concentrations were calculated based on GC peak areas without using correction factors. Pure commercial essential oil components used as standards for GC-FID analyses were obtained from Aldrich and Fluka.

## 4. Conclusions

Among the volatile compounds found in *C. europaea* several have semiochemical and antimicrobial activities. As regards to semiochemicals they may play a role in the defence of the plant against herbivores by discouraging foraging on stems and fruits [1]. It is interesting to note that, as reported by Dudareva *et al.* [1], some volatiles may not always be beneficial to the plant. This is the case of the four pheromones which attract *Danaus chrysippus* to oviposit on fruits and to feed on stems and fruits. These pheromones play a role as kayromones at least in these combination of species: *C. europaea* – *D. chrysippus*. The presence of antimicrobials could increase the fitness of the plant by arresting the spread of pathogens. Further investigations on *C. europaea* essential oils would be interesting to test the actual antimicrobial potential and to verify also its pharmaceutical interest.

## Figures and Tables

**Table 1:** Percent composition of the essential oils of stems (S) and fruits (F) of *Caralluma europaea* (Guss) N.E.Br.

K_i1_^a^	K_i2_^b^	Ident.^c^	Compounds	S %	F %
800	800	1, 2, 3	Octane	0.3	
901		1, 2	Heptanal		t
936	1075	1, 2, 3	α-Pinene		0.4
963	1543	1, 2, 3	Benzaldehyde		0.3
980	1454	1, 2	1-Octen-3-ol		0.6
1001		1, 2	Octanal		0.1
1002		1, 2	2-Pentylfuran		0.1
1044	1663	1, 2, 3	Phenylacetaldehyde		0.6
1058	1657	1, 2, 3	Acetophenone		t
1097		1, 2	Methyl benzoate		0.1
1102		1, 2	Nonanal		t
1167	1734	1, 2	4-Ethyl benzaldehyde		
1179	1763	1, 2, 3	Naphtalene	t	
1197		1, 2	Safranal		0.2
1206	1508	1, 2	Decanal		0.4
1208		1, 2	α-Ionene	0.7	t
1212		1, 2	β-Cyclocitral		0.1
1243		1, 2	1,2,3,4-tetrahydro-1,5,7-trimethylnaphthalene		t
1261		1, 2	(*E*)-2-Decenal		0.3
1291	2471	1, 2, 3	Indole	0.6	
1306		1, 2	Undecanal		0.1
1313	2180	1, 2	4-Vinylguaiacol		0.4
1342		1, 2	1,2-Dihydro-1,1,6-trimethylnaphthalene		0.5
1349		1, 2	Dehydro-ar-ionene		0.2
1358	1787	1, 2	(*E*)- β-Damascenone		0.3
1382		1, 2	β-Cubebene		t
1409		1, 2	2-Ethyl-1,4-dimethylbenzene		0.2
1410		1, 2	Methyl indole	t	
1415	1722	1, 2	Dodecanal		0.7
1450	1621	1, 2	Widdrene	4.9	
1470		1, 2	2,5-Cyclohexadiene-1,4-dione, 2,6-bis(1,1-dimethylethyl)		0.3
1509	1746	1, 2	(*Z*)-α-Bisabolene	1.2	
1578	2150	1, 2	Spathulenol		1.5
1651	2253	1, 2	β-Eudesmol	1.9	5.4
1659		1, 2	Valerenol	1.2	
1674		1, 2	Tetradecanol	0.4	
1758	2713	1, 2, 3	Tetradecanoic acid	5.6	
1771		1, 2	Pentadecanol		0.3
1792		1, 2	1-Octadecene	0.6	
1819	2135	1, 2	Hexadecanal		0.7
1845	2131	1, 2	Hexahydrofarnesylacetone	3.8	2.8
1892		1, 2	1-Nonadecene	0.4	
1900	1900	1, 2, 3	Nonadecane	0.8	
1950	2622	1, 2	(*Z*)-Phytol	1.7	
1957		1, 2, 3	Hexadecanoic acid ethyl ester		0,7
1958	2931	1, 2, 3	Hexadecanoic acid	7.8	9.6
1992		1, 2	1-Eicosene	0.6	
2000	2000	1, 2, 3	Eicosane	0.9	
2023	2354	1, 2	Octadecanal	1.1	
2082	2597	1, 2	Octadecanol		0.2
2100	2100	1, 2, 3	Heneicosane	2.5	3.2
2102		1, 2	2-Nonadecanone	0.4	
2104	3160	1, 2, 3	(*Z,Z*)-9,12-Octadecadienoic acid	5.2	2.4
2132	2625	1, 2	(*E*)-Phytol	2.6	3.9
2194		1, 2	1-Docosene	0.2	
2200	2200	1, 2, 3	Docosane	0.4	1.4
2300	2300	1, 2, 3	Tricosane	4.4	7.3
2400	2400	1, 2, 3	Tetracosane	1.7	1.9
2452		1, 2	Docosanol	0.4	
2493		1, 2	1-Pentacosene	2.1	
2500	2500	1, 2, 3	Pentacosane	5.4	6.5
2594		1, 2	1-Hexacosene		2.1
2600	2600	1, 2	Hexacosane		2.9
2630		1, 2	Tetracosanal		0.9
2658		1, 2	1-Tetracosanol		0.4
2700	2700	1, 2	Heptacosane	6.1	9.9
2728		1, 2	Hexacosanal		1.9
2793		1, 2	1-Octacosene		1.8
2800	2800	1, 2	Octacosane	2.4	0.8
2827		1, 2	Squalene	1.2	1.2
2900	2900	1, 2	Nonacosane	8.3	6.5
3100	3100	1, 2	Hentriacontane	9.5	7.7
3200	3200	1, 2	Dotriacontane	0.9	0.3
3300	3300	1, 2	Tritriacontane	1.4	1.4
			**Total compounds**	**89.7**	**91.5**

^a^ K_i1_: HP 5MS column; ^b^ K_i2_: HP Innowax column; ^c^ Ident.: 1 = retention index identical to bibliography; 2 = identification based on comparison of MS; 3 = retention time identical to authentic compounds; t: trace amount < 0.05%.

**Table 2:** Essential oils of the stems and fruits of *Caralluma europaea* (Guss) N.E.Br. arranged by class.

Compounds	Semiochemicals^a^					Antimicrobial^b^	Apocynaceae^c^
	A	Al	P	K	Sy		
Aromatic Compounds							
Aldehydes							
Benzaldehyde	x	x	x	x			LC [12,13]SC [13]
Phenylacetaldehyde	x	x	x	x			FH [14]LC [13]SC [13]
4-Ethylbenzaldehyde						x	
**Hydrocarbons**							
Naphtalene	x		x	x			
α-Ionene							
1,2,3,4-tetrahydro-1,5,7-trimethylnaphthalene							
1,2-Dihydro-1,1,6-trimethylnaphthalene							
Dehydro-ar-ionene							
2-Ethyl-1,4-dimethylbenzene							
**Esters**							
Methyl benzoate	x		x	x		x	FH [14]
**Oxygen containing compounds**							
2-Pentylfuran							
**Ketones**							
Acetophenone	x	x	x	x		x	
2,5-Cyclohexadiene-1,4-dione,2,6-bis(1,1-dimethyl-ethyl)							
**Phenolic compounds**							
4-Vinylguaiacol						x	
**Nitrogen containing compounds**							
Indole	x	x	x	x		x	LC [13]
Methyl indole							
**Non-Aromatic Compounds**							
**Hydrocarbons**							
Octane	x			x			
1-Octadecene		x		x			
1-Nonadecene		x		x			
Nonadecane	x	x		x			LC [12]
1-Eicosene				x			
Eicosane			x	x			LC [12]
Heneicosane	x			x			
1-Docosene				x			
Docosane		x	x	x			LC [12]
Tricosane	x	x	x	x			LC [12]
Tetracosane	x	x	x	x			LC [12]
1-Pentacosene				x			
Pentacosane	x	x	x	x			
1-Hexacosene				x			
Hexacosane	x	x		x			
Heptacosane		x		x			
1-Octacosene		x					
Octacosane		x		x			
Nonacosane	x	x		x			
Hentriacontane				x			
Dotriacontane				x			
Tritriacontane				x			
**Alcohols**							
1-Octen-3-ol	x	x	x	x			FH [14]
Tetradecanol	x			x		x	
Pentadecanol				x		x	
Octadecanol				x			FH [14]
Docosanol				x			
1-Tetracosanol							
**Aldehydes**							
Heptanal	x	x	x	x			
Octanal	x	x	x	x		x	LC [13]SC [13]
Nonanal	x	x	x	x		x	LC [12]FH [14]
Decanal	x	x	x	x		x	LC [12]FH [14]
(*E*)-2-Decenal	x	x	x	x			LC [12,13]SC [13]FH [14]
Undecanal	x	x		x		x	LC [12]
Dodecanal		x	x	x			LC [12]
Hexadecanal	x	x		x		x	FH [14]
Octadecanal				x		x	
Tetracosanal							
Hexacosanal							
**Ketones**							
(*E*)-α-Damascenone							
Hexahydrofarnesylacetone				x			LC [13]SC [13]
2-Nonadecanone							
**Monoterpene Hydrocarbons**							
α-Pinene	x	x	x	x		x	
Oxygenated Monoterpenes								
Safranal							LC [13]	
α-Cyclocitral							LC [13]
**Sesquiterpene hydrocarbons**								
β-Cubebene							
Widdrene							
(*Z*)-α-Bisabolene							
**Oxygenated sesquiterpenes**								
Spathulenol	x					x	
β-Eudesmol		x					
Valerenol							
**Fatty acids**								
Tetradecanoic acid		x		x		x	LC [12]
Hexadecanoic acid	x	x	x	x		x	LC [12]
(*Z,Z*)-9,12-Octadecadienoic acid	x	x	x	x		x	LC [12]
**Esters**								
Hexadecanoic acid ethyl ester				x			LC [13]SC [13]
**Diterpenes**								
(*Z*)-Phytol						x	LC [13]
(*E*)-Phytol		x		x		x	
**Triterpenes**								
Squalene	x	x		x		x	

^a^ Semiochemicals: (A: Attractant; Al: Allomone; K: Kairomone; P: Pheromone; Sy: Synomone) [15]. ^b^ Antimicrobial: Antimicrobial Data of Drugs, Natural Compounds and Essential Oils [16]. ^c^ Aerial parts of other Apocynaceae: LC: Leaves of Catharanthus roseus [12,13]; SC: Stems of Catharanthus roseus [13]; FH: Fruits of Hancornia speciosa [14].
